# Systemic inflammatory response markers improve the discrimination for prognostic model in hepatocellular carcinoma

**DOI:** 10.1007/s12072-025-10806-6

**Published:** 2025-03-25

**Authors:** Alba Rocco, Costantino Sgamato, Filippo Pelizzaro, Vittorio Simeon, Pietro Coccoli, Debora Compare, Elisa Pinto, Giorgio Palano, Francesco Giuseppe Foschi, Giovanni Raimondo, Gabriele Missale, Gianluca Svegliati-Baroni, Franco Trevisani, Eugenio Caturelli, Maurizia Rossana Brunetto, Gianpaolo Vidili, Alberto Masotto, Donatella Magalotti, Claudia Campani, Antonio Gasbarrini, Francesco Azzaroli, Gian Ludovico Rapaccini, Bernardo Stefanini, Rodolfo Sacco, Andrea Mega, Edoardo Giovanni Giannini, Giuseppe Cabibbo, Mariella Di Marco, Maria Guarino, Paolo Chiodini, Fabio Farinati, Gerardo Nardone

**Affiliations:** 1 Department of Clinical Medicine and Surgery, Gastroenterology Unit, University Federico II, Via S. Pansini N° 5, 80131 Naples, Italy; 2 Department of Surgery, Oncology and Gastroenterology, Gastroenterology Unit, University of Padova, Padua, Italy; 3 Department of Surgery, Oncology and Gastroenterology, Gastroenterology Unit, Azienda Ospedaliero-Universitaria of Padova, Padua, Italy; 4 Medical Statistics Unit, University of Campania “Luigi Vanvitelli”, Naples, Italy; 5 Department of Internal Medicine, Ospedale Per Gli Infermi Di Faenza, IRCCS Meldola, AUSL Romagna, Faenza, Italy; 6 Department of Clinical and Experimental Medicine, Clinical and Molecular Hepatology Unit, University of Messina, Messina, Italy; 7 Department of Medicine and Surgery, Unit of Infectious Diseases and Hepatology, University of Parma, Parma, Italy; 8 Gastroenterology Unit, Polytechnic University of Marche, Ancona, Italy; 9 Unit of Semeiotics, Liver and Alcohol-Related Diseases, Department of Medical and Surgical Sciences, IRCCS Azienda Ospedaliero-Universitaria Di Bologna, Bologna, Italy; 10 Department of Medical and Surgical Sciences, University of Bologna, Bologna, Italy; 11 Gastroenterology Unit, Belcolle Hospital, Viterbo, Italy; 12 Department of Clinical and Experimental Medicine, Hepatology and Liver Physiopathology Laboratory and Internal Medicine Unit, University of Pisa, Pisa, Italy; 13 Department of Medicine Surgery and Pharmacy, Centralized Day Hospital of the Medical Area, University of Sassari, Azienda Ospedaliero-Universitaria Di Sassari, Sassari, Italy; 14 Gastroenterology Unit, Ospedale Sacro Cuore Don Calabria, Negrar, Italy; 15 Division of Internal Medicine, Neurovascular and Hepatometabolic Diseases, IRCCS Azienda Ospedaliero-Universitaria Di Bologna, Bologna, Italy; 16 Department of Experimental and Clinical Medicine, Internal Medicine and Hepatology Unit, University of Firenze, Florence, Italy; 17 Internal Medicine and Gastroenterology, Fondazione Policlinico Universitario Agostino Gemelli IRCCS, Università Cattolica del Sacro Cuore, Rome, Italy; 18 Gastroenterology Unit, Department of Surgical and Medical Sciences, IRCCS Azienda Ospedaliero-Universitaria Di Bologna, Bologna, Italy; 19 Division of Internal Medicine, Hepatobiliary and Immunoallergic Diseases, IRCCS Azienda Ospedaliero-Universitaria Di Bologna, Bologna, Italy; 20 Gastroenterology and Digestive Endoscopy Unit, Foggia University Hospital, Foggia, Italy; 21 Gastroenterology Unit, Bolzano Regional Hospital, Bolzano, Italy; 22 Gastroenterology Unit, Department of Internal Medicine, University of Genova, Genoa, Italy; 23 IRCCS Ospedale Policlinico San Martino, Genoa, Italy; 24 Department of Health Promotion, Mother and Child Care, Internal Medicine and Medical Specialties, PROMISE, Gastroenterology and Hepatology Unit, University of Palermo, Palermo, Italy; 25 Medicine Unit, Bolognini Hospital, Seriate, Italy; 26 Department of Clinical Medicine and Surgery, Diseases of the Liver and Biliary System Unit, University of Naples “Federico II”, Naples, Italy

**Keywords:** Neutrophil, To, Lymphocyte ratio, Platelet, To, Lymphocyte ratio, Survival, Recurrence, Free survival, Hepatocellular carcinoma, Prognosis

## Abstract

**Background/purpose of the study:**

We aimed to evaluate the performance of neutrophil-to-lymphocyte ratio (NLR), platelet-to-lymphocyte ratio (PLR), and their combination (combined NLR-PLR, CNP) in predicting overall survival (OS) and recurrence-free survival (RFS) in a large cohort of unselected hepatocellular carcinoma (HCC) patients.

**Methods:**

Training and validation cohort data were retrieved from the Italian Liver Cancer (ITA.LI.CA) database. The optimal cut-offs of NLR and PLR were calculated according to the multivariable fractional polynomial and the minimum *p* value method. The continuous effect and best cut-off categories of NLR and PLR were analyzed using multivariable Cox regression analysis. A shrinkage procedure adjusted over-fitting hazard ratio (HR) estimates of best cut-off categories. C-statistic and integrated discrimination improvement (IDI) were calculated to evaluate the discrimination properties of the biomarkers when added to clinical survival models.

**Results:**

2,286 patients were split into training (*n* = 1,043) and validation (*n* = 1,243) cohorts. The optimal cut-offs for NLR and PLR were 1.45 and 188, respectively. NLR (HR 1.58, 95% CI 1.11–2.28, *p* = 0.014) and PLR (HR 1.79, 95% CI 1.11–2.90, *p* = 0.018) were independent predictors of OS.

When incorporated into a clinical prognostic model that includes age, alpha-fetoprotein (AFP), the CHILD–Pugh score, and the Barcelona Clinic Liver Cancer (BCLC) staging system, CNP had a significant incremental value in predicting OS (IDI 1.3%, *p* = 0.04). Data were confirmed in the validation cohort. Neither NLR nor PLR significantly predicted RFS in the training cohort.

**Conclusions:**

NLR, PLR, and CNP independently predicted shorter OS in HCC patients. The addition of CNP to the survival prediction model significantly improved the model’s accuracy in predicting OS.

**Graphical Abstract:**

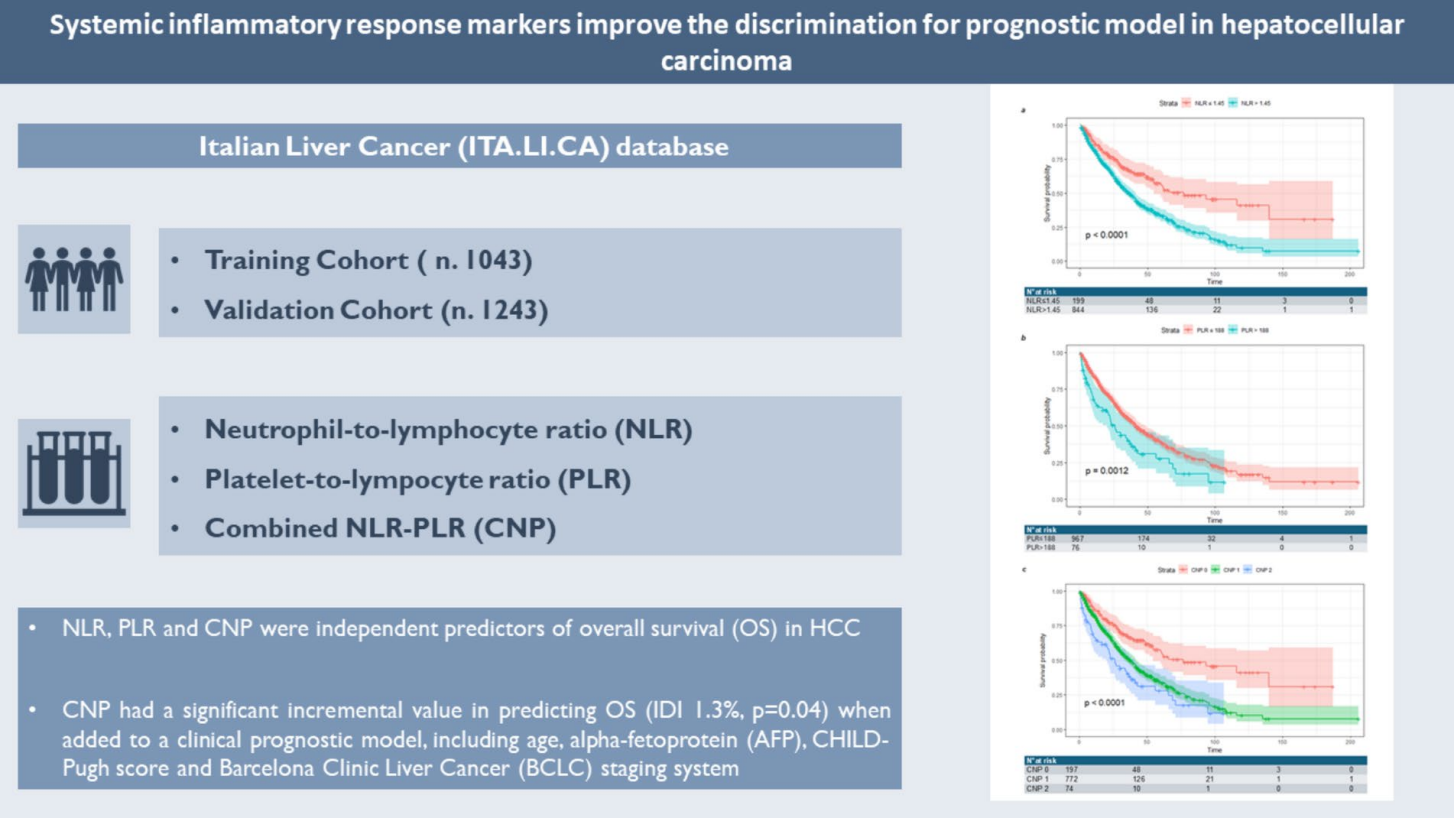

**Supplementary Information:**

The online version contains supplementary material available at 10.1007/s12072-025-10806-6.

## Introduction

Hepatocellular carcinoma (HCC) is the seventh most common cancer and the second leading cause of cancer-related deaths worldwide. The overlap between incidence and mortality (830,000 deaths per year) reflects the dismal prognosis of this disease, likely due to the late diagnosis and the high recurrence rate [1]. Accurate prognostic evaluation of patients with HCC is essential to optimize treatment strategies and improve the outcomes. However, stratifying death risk can be challenging because HCC is a unique malignancy that typically develops in the setting of pre-existing liver disease (primarily cirrhosis), thus raising the competing risk of dying from cancer progression and liver decompensation [2].

Over the last 20 years, several prognostic systems have been introduced, mainly based on variables commonly accepted as survival-associated factors in patients with HCC, such as tumor burden, residual liver function, and general clinical conditions (performance status) [3]. Alpha-fetoprotein (AFP) is the only serum biomarker included in some of these models despite its suboptimal accuracy as a prognostic indicator [4]. Therefore, it is a compelling argument to identify other potential non-invasive biomarkers to refine the patient’s prognosis.

In recent years, increasing evidence has demonstrated that the systemic inflammatory response (SIR) plays a crucial role in the pathogenesis and progression of liver cirrhosis as well as in the development and advancement of different types of cancer [5, 6].

Circulating SIR markers, including the neutrophil–lymphocyte ratio (NLR), platelet-lymphocyte ratio (PLR), or their combination (combined NLR and PLR: CNP) reflect the balance between pro-tumor inflammation associated with platelets and neutrophils and the anti-tumor immune response mediated by lymphocytes. These convenient, easily obtainable and repeatable biomarkers have been proven to have prognostic significance in patients with different cancers [7–9]. However, studies analyzing the prognostic accuracy of serum SIR markers in HCC patients do not allow definitive conclusions on their utility in clinical practice [10–14].

Based on these premises, we aimed to analyze the prognostic accuracy of NLR, PLR, and CNP in a large cohort of Caucasian patients with HCC included in the Italian Liver Cancer (ITA.LI.CA.) database.

## Material and methods

### Patients

The ITA.LI.CA registry currently includes 7782 patients consecutively diagnosed with HCC and managed in 24 Italian centres from January 1987 to December 2022. Participating institutions prospectively collect clinical data about patients with HCC and update them every two years. The group coordinator (F.T., Bologna University) regularly checks entries for consistency. Whenever a clarification or additional information is deemed necessary, relevant cases are returned to the recruiting centre before the final inclusion. The ITA.LI.CA database management conforms to the past and current Italian legislation regarding privacy, and the present study conforms to the ethical guidelines of the Declaration of Helsinki. The study was approved by the Institutional Review Board of the ITA.LI.CA coordinating centre, Alma Mater Studiorum University of Bologna, on 15th May 2012 (approval number 99/2012/O/Oss). Written informed consent was obtained from all participants.

In the ITA.LI.CA registry, demographic data, etiology of liver disease, and comorbidities are systematically collected. From the entire database, we selected patients who were diagnosed with HCC for the first time and were treatment-naive during the period from January 2000 and December 2022 with available pertinent laboratory data at the time of the first HCC treatment and a lag time of less than six months between HCC diagnosis and treatment. Exclusion criteria were as follows: concomitant severe comorbidities (advanced renal, cardiac, pulmonary, and/or thyroid disorders), and incomplete data.

Data from 1,043 HCC patients (from January 2000 to December 2018) and 1,243 HCC patients (from January 2019 to December 2022) were reviewed as training and internal validation cohorts, respectively. Treatment of chronic viral hepatitis was performed according to current guidelines at the time of enrolment [15–19]. Diagnosis of liver cirrhosis was defined at the time of enrolment based on the combination of laboratory and imaging tests. The Child–Pugh class, Model for End-Stage Liver Disease (MELD) score, ascites and hepatic encephalopathy, and clinically significant portal hypertension (CSPH) were also recorded. Since hepatic venous pressure gradient (HVPG) measurement, the gold standard method for diagnosing CSPH is not generally performed in clinical practice, the diagnosis was based on unequivocal signs of CSPH (splenomegaly, esophageal varices, ascites, and platelet count ≤ 150 × 10^9^/L).

Laboratory data, such as white blood cell (WBC) count, neutrophil count, lymphocyte count, platelet (PLT) count, international normalized ratio (INR), albumin, total bilirubin, creatinine, and AFP are regularly registered in the database. The prognostic cut-off value of AFP was arbitrarily set at 400 ng/mL. For our study, we analysed laboratory data obtained at the time of the first HCC diagnosis.

### SIR biomarkers definition

The NLR was calculated as absolute neutrophil count (the number of neutrophils/μL) divided by absolute lymphocyte count (number of lymphocytes/μL). The PLR was calculated as absolute platelet count (the number of platelets/μL) divided by absolute lymphocyte count (number of lymphocytes/μL).

### Diagnosis and staging of HCC

The diagnosis of HCC was made according to the guidelines available at the time of inclusion. Among the patients selected for this study, the diagnosis of HCC was confirmed by histology in 211 out of 1043 (20%) cases in the training cohort and in 235 out of 1243 (19%) cases in the validation cohort. In the remaining cases the diagnosis of HCC was  based on radiologic criteria at imaging (4-phase multidetector computed tomography [CT]) or dynamic contrast-enhanced magnetic resonance [MRI]. Dynamic imaging techniques assessed the location, size and number of nodules, macrovascular invasion (MVI), and extrahepatic spread (EHS). HCC was staged according to Barcelona Clinic Liver Cancer (BCLC) [20] and ITA.LI.CA staging systems [21]. The diagnosis of HCC recurrence was similar to that of the initial disease diagnosis.

### Treatment and follow-up

Treatments for HCC, including liver transplantation (LT), liver resection (LR), percutaneous radiofrequency ablation or other ablative techniques (ABL), transarterial chemoembolisation/embolisation or radioembolisation (IAT, intra-arterial therapies), systemic therapies (mainly sorafenib, SOR) and best supportive care (BSC) were performed according to BCLC criteria.

Overall survival (OS) was defined as the time interval from treatment to the date of death, last follow-up, or data censoring (i.e. 31st December 2018 for the training cohort and 31st December 2022 for the validation cohort). Recurrence-free survival (RFS) was defined as the time interval between treatment and the first evidence of disease recurrence, excluding cases of HCC detected within three months following treatment. It was evaluated in the subset of patients who underwent potentially curative therapy, namely LT, LR, and ABL.

### Statistical analysis

Continuous variables were described using mean and standard deviation (SD) or median and interquartile range (IQR) according to their distribution. The differences were compared using the Student’s t-test and the Mann–Whitney U test. Categorical variables were reported as the number of cases and percentages, and the differences were compared using the Chi-square test. A histogram graphically described the distribution of NLR and PLR, whereas a scatterplot and the Spearman rho coefficient highlighted the correlation between biomarkers.

The associations between NLR and PLR and clinical factors or staging systems known to be related to HCC prognosis, such as age at diagnosis, AFP, BCLC stage and Child–Pugh score, were analyzed using the Wilcoxon Rank Sum and Signed Rank Tests for dichotomous variables and the Kruskal–Wallis Rank Sum Test for categorical variables.

The Cox proportional hazard model was used to test the prognostic role of NLR and PLR on both OS and RFS. In the first univariate analysis, NLR and PLR were tested as continuous variables after the linearity assumption was tested using fractional polynomials. In a second univariate analysis, NLR and PLR were tested as categorical variables dichotomized according to the best cut-off value that minimizes the p-value of hazard ratio (HR). According to the identified cut-offs, OS and RFS curves were estimated using the Kaplan–Meier method and compared with a two-sided log-rank test.

For each biomarker (and for both continuous effect and best cut-off categories), a multivariable analysis was performed using covariates: age, AFP, BCLC stage and Child–Pugh score. A shrinkage procedure with 95% confidence interval (CI) was calculated using the bootstrap-percentile method to adjust for over-fitting HR estimates of best cut-off categories [22].

To evaluate the incremental clinical value and the discrimination properties of SIR biomarkers, we compared the basic clinical model with those including biomarkers (continuous or categorical) using C-statistic and integrated discrimination improvement (IDI) measures. Data were analyzed using R software version 4.1.2 (R Foundation for Statistical Computing, Vienna, Austria).

## Results

### Demographic and tumor characteristics of the enrolled population

**Fig. 1:**
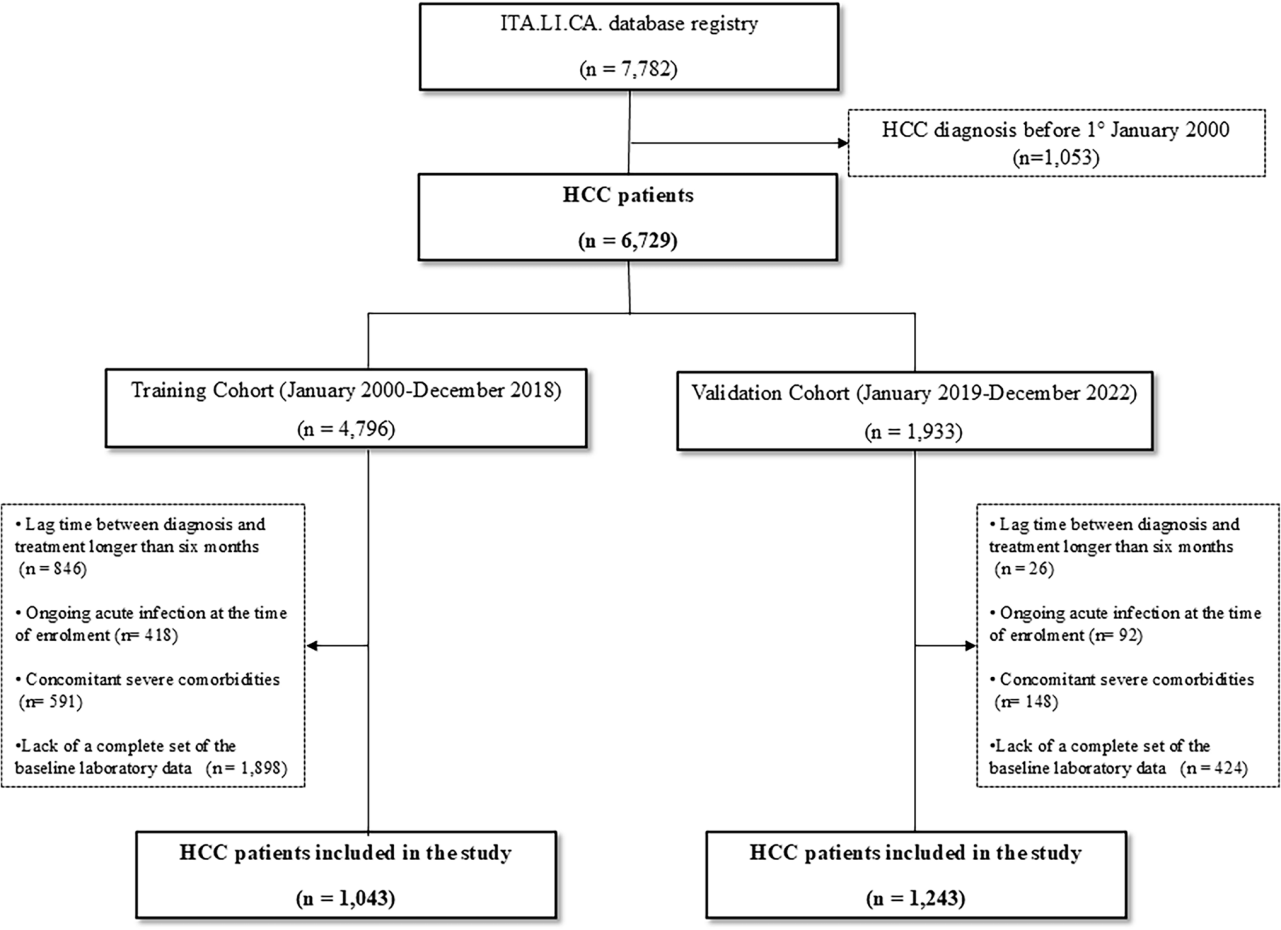
Flow-chart of the study

A total of 2,286 patients who met the eligibility criteria were enrolled. One thousand forty-three were included in the training cohort and 1,243 in the validation cohort. The flow chart of the study is shown in Fig. 1.

The baseline demographic, clinical, and laboratory findings of HCC patients are summarised in Table 1.

**Table 1:** Baseline demographic, clinical and laboratory findings, and HCC characteristics in the studied population

	Training Cohort (*n* = 1043)	Validation Cohort (*n* = 1243)
***Patient factors***		
Males	813 (78)	992 (80)
Age (years)	69 [60–75]	68.8 [60.6—76.5]
BMI (kg/m^2^)	25.8 [23–29]	26.0 [23.4—29.1]
***Liver disease characteristics***		
Etiology (n, %)		
Viral	700 (67)	518 (42)
Non-viral	97(9)	500 (40)
Combo	246 (24)	225 (18)
Cirrhosis (n, %)	889 (88)	1003(81)
CRPH (n, %)	733 (76)	658 (53)
Child–Pugh class (n, %)		
A	622 (70)	687 (68)
B	240 (27)	268 (27)
C	27 (3)	48 (5)
MELD	9 [8–12]	9.1 [8–12]
ECOG-PS > 0	252 (24)	335 (27)
***Laboratory data***		
Albumin (gr/dL)	3.7 [3.3–4]	3.8 [3–4]
Total serum bilirubin (mg/dL)	1 [0.7–1.6]	0.94 [0.6–1.5]
INR	1.2 [1.1–1.3]	1.14 [1.1–1.3]
Creatinine (mg/dL)	0.8 [0.7–1]	0.86 [0.7–1.]
Na + (mmol/dL)	139 [137–141]	139 [137–141]
K + (mmol/dL)	4.1 [3.8–4.4]	4.2 [3.9–4.5]
AFP (µg/L)	9 [4–44]	8.1 [3.5–87]
Hb (gr/dL)	13 [11.6–14.4]	13 [11.4–14.3]
WBC (10^3^ cells/mm^3^)	5.1 [4–6.5]	5.7 [4.3–7.7]
Neutrophiles (10^3^ cells/mm^3^)	3[2.2–4.1]	3.4 [2.4–4.6]
Lymphocytes (10^3^ cells/mm^3^)	1.31 [1–1.8]	1.35 [1–1.8]
PLT (10^3^ cells/mm3)	109 [77–161]	134 [87–194]
***Tumor characteristics***		
Single tumor (n,%)	700 (67)	1032 (83)
MTD (mm, median [IQR])	30 [20–43]	30 [20–55]
MVI (n, %)	246 (24)	192(15)
EHS (n, %)	45(5)	102(8)
BCLC stage (n, %)		
0	154 (15)	60 (5)
A	439 (42)	610 (49)
B	154 (15)	141 (11)
C	249 (24)	360 (29)
D	47 (4)	72 (6)
ITA.LI.CA stage(n, %)		
0	233(22)	219 (18)
A	429 (41)	367 (29)
B	227 (22)	448 (36)
C	154 (15)	209 (17)
***Main treatment (n, %)***		
LT	29 (3)	112 (9)
LR	194 (19)	281 (23)
ABL	348 (33)	360 (29)
IAT	329 (31)	269 (22)
SOR	86 (8)	116 (9)
BSC	57 (6)	105 (8)

Data are described as number (%) or median [IQR]

*BMI* body mass index, *CRPH* clinically relevant portal hypertension, *MTD* maximal tumor diameter, *MVI* macrovascular invasion, *EHS* extrahepatic spread, *LT* liver transplantation, *LR* liver resection, *ABL* percutaneous ablation, *IAT* intra-arterial therapies, *SC* systemic chemotherapy, *BSC* best supportive care

### Inflammatory markers and association with clinical prognostic factors

The median NLR and PLR were 2.22 (IQR 1.63–3.18) and 87 (IQR 58–124). Both biomarkers showed a skewed distribution and were positively correlated (rho = 0.56) (Supplementary Fig. 1).

As far as concerned the association between NLR and PLR and clinical prognostic factors (age, AFP, Child–Pugh score and BCLC stage), PLR correlated with age [PLR rho: 0.15, *p* < 0.001], and its median value was significantly lower in patients with AFP > 400 ng/mL than in those with AFP < 400 ng/ml (84 [IQR 56–119] vs 96 [IQR 65–133], *p* = 0.002). NLR was associated with the Child–Pugh score (*p* < 0.001), whereas both biomarkers were associated with BCLC staging, progressively increasing with tumor burden *(p* < 0.001 and *p* < 0.001 for NLR and PLR, respectively (Supplementary Table 1).

### SIR biomarkers are independent prognostic factors of OS

**Fig. 2:**
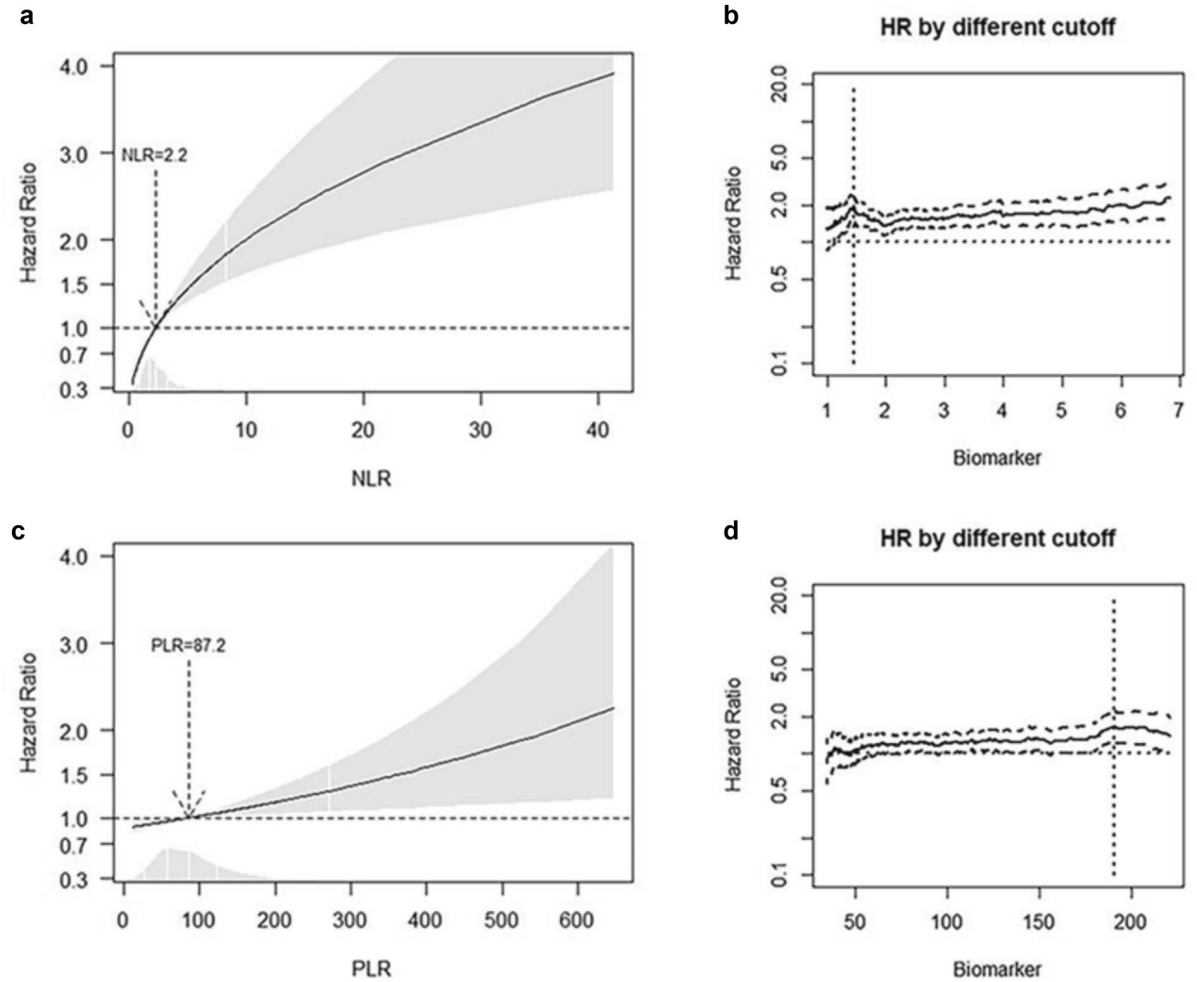
Continuous association and best cut-off of NLR and PLR biomarkers. Smoothed graph of the association of NLR (panel **a**) and PLR (panel **c**) following evaluation of the linearity assumption using fractional polynomials; note the logarithmic shape of NLR. The best cut-off value minimizes the *p* value of the HR for NLR (panel **b**) and PLR (panel **d**). Value crossing HR = 1 represents the median value for each biomarker

According to fractional polynomial analysis, NLR was associated with OS as a logarithmic function [HR 1.61 (95% CI 1.39–1.86, *p* < 0.001)] while PLR was linearly associated with OS [HR 1.16 (95% CI 1.04–1.29, *p* < 0.01)] (Fig. 2, panel A & C). The best cut-offs minimizing the p-value of HR in the training cohort were 1.45 for NLR and 188 for PLR, respectively (Fig. 2, panels b & d).

Eight hundred and forty-four patients (81.4%) had NLR > 1.45, and 76 (7.3%) patients had PLR > 188.

In the univariate model, both biomarkers were associated with OS irrespective of shrinkage procedure (Shrunken coefficients: NLR HR 1.86, 95% CI 1.40–2.48, *p* < 0.001, and PLR HR 1.57, 95% CI 1.08–2.30, *p* = 0.019, respectively). In the multivariate model adjusted for the clinical covariates reported to be prognostic (age, AFP, Child–Pugh score, and BCLC stage), both NLR and PLR were significantly associated with OS (NLR: HR 1.65, 95% CI 1.18–2.29, *p* = 0.003, and PLR: HR 1,87, 95% CI 1.24–2.81, *p* = 0.003). By applying a shrinkage procedure to adjust for over-fitting HR estimates, both NLR > 1.45 (HR 1.58, 95% CI 1.11–2.28, *p* = 0.014) and PLR > 188 (HR 1.79, 95% CI 1.11–2.90, *p* = 0.018) remained associated with OS (Table 2).

**Table 2:** Analysis of biomarkers best cut-off for OS

	Original			Shrunken coefficients Bootstrap CI		
	HR	(95% CI)	*p*	HR	(95% CI)	*p*
NLR cut-off > 1.45						
Unadjusted model	1.91	(1.45–2.52)	< 0.0001	1.86	(1.40–2.48)	< 0.001
Adjusted model	1.65	(1.18–2.29)	0.003	1.58	(1.11–2.28)	0.014
PLR cut-off > 188						
Unadjusted model	1.64	(1.20–2.24)	0.002	1.57	(1.08–2.30)	0.019
Adjusted model	1.87	(1.24–2.81)	0.003	1.79	(1.11–2.90)	0.018

*NLR* neutrophil-to-lymphocyte ratio, *PLR* platelet-to-lymphocyte ratio, *HR* hazard ratio, *CI* confidence interval

An inflammation-based score was created by combining NLR with PLR (combined NLR-PLR score, CNP) and analyzed as a continuous and dichotomous variable after identifying the best cut-off. Patients were then classified as follows: those with both NLR and PLR below their respective cut-off entered in the CNP = 0 group (*n* = 197, 19%), those with either NLR or PLR above the cut-off were classified in the CNP = 1 group (*n* = 772, 74%), and those with both NLR and PLR above their respective cut-off were classified in the CNP = 2 group (*n* = 74, 7%).

### Survival analysis

When the study was censored (31st December 2022), the median follow-up times were 40 and 27 months in the training and validation cohorts, respectively. The median OS for each treatment was as follows: 31 [20–83] months for LT, 68 [51—68] months for LR, 55 [48–65] months for ABL, 33 [28–39] months for IAT, 7 [6–9.7] months for SOR, and 3 [2–5] months for BSC.

**Fig. 3:**
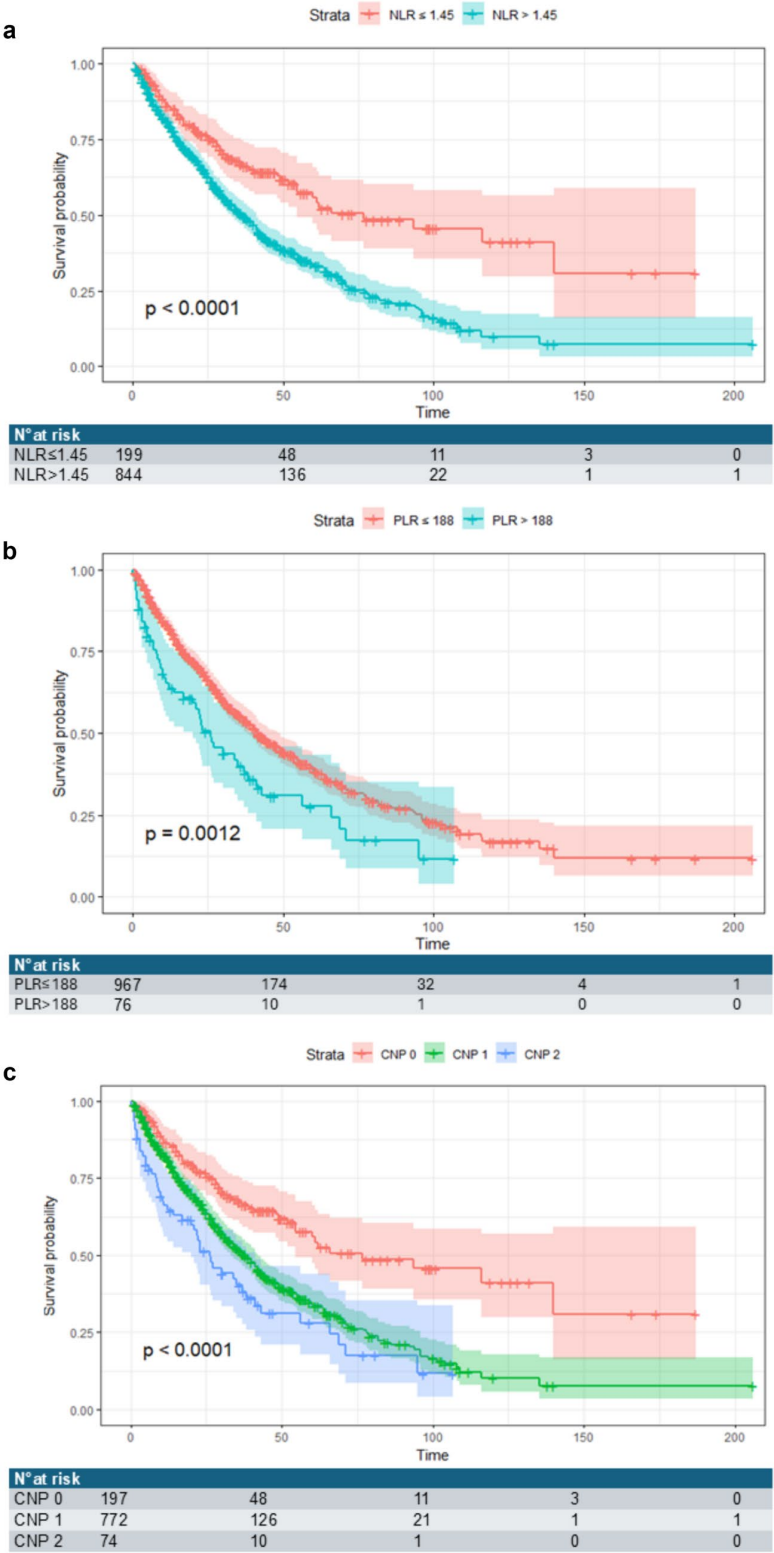
Kaplan–Meier curves of survival data of HCC patients (*n* = 1,043) according to NLR (**a**) and PLR (**b**) best cut-offs and CNP (**c**). Time on the x-axis represents months of observation (follow-up extended up to 206 months). The table with subjects at risk is reported for each biomarker at each specific time point. P value is a log-rank test

Figure 3 shows the survival trees of OS in the training cohort according to the different SIR markers. Median OS time was significantly higher in patients with NLR and PLR below the cut-off values (*p* < 0.0001 for NLR and *p* = 0.00015 for PLR, respectively) (Fig. 3, panels a and b). Likewise, CNP stratified patients according to their prognosis since median OS was significantly lower in CNP 2 [26 months (95% CI 20–41)] than in CNP 1 [38 months (95% CI 33–42)] or CNP 0 [76.8 months, 95%CI 54.6- not achieved] patients (*p* < 0.0001) (Fig. 3, panel *C*).

CNP was a significant predictor of OS, irrespective of the underlying etiology of liver disease (*p* < 0.001 for viral etiology, *p* = 0.047 for non-viral etiology, and *p* = 0.042 for combined etiology). Finally, CNP significantly predicted OS in the subgroups of patients undergoing ABL (*p* = 0.037) and IAT (*p* = 0.011), whereas it did not predict OS in the subgroups of patients who underwent LR (*p* = 0.51), SOR (*p* = 0.27), and BSC (*n* = 57, *p* = 0.63).

Similar results were observed in the validation cohort (Supplementary Fig. 2). Patients with NLR and PLR below the cut-off values had significantly higher median OS time (*p* < 0.001 for NLR and *p* < 0.001 for PLR, respectively) (Supplementary Fig. 2, panels A and B). CNP was able to stratify patients according to their prognosis since median OS was significantly lower in CNP 2 [18 months (95% CI 12.9–38.5)] than in CNP 1 [45.3 months (95% CI 31.8–NA)] or CNP 0 [NA months, 95%CI 41.4–NA] patients (*p* < 0.0001) (Supplementary Fig. 2, panel C).

### Discrimination properties of SIR biomarkers

Three different models were fitted to evaluate the potential role of NLR, PLR, and CNP in predicting survival, together with established prognostic factors. The basic model included standard prognostic factors: age, AFP, Child–Pugh score, and the BCLC staging system. The second model included all the above-mentioned variables plus the biomarkers (log-transformed NLR and PLR) considered as continuous variables, with tests for possible interactions. The third model replaced the continuous biomarkers with the CNP score. This progression of models allows for a comparison of predictive accuracy and interpretability by assessing if the addition of serum SIR biomarkers (in continuous or composite form) significantly enhances survival prediction beyond traditional prognostic factors.

The results are shown in Table 3. Log NLR (HR 1.33, 95% CI 1.09–1.62, *p* = 0.004) and CNP (HR 2.69, 95% CI 1.79–4.06, *p* < 0.001) were significantly associated with OS.

**Table 3:** Comparison of discrimination for a new model including baseline prognostic factors and SIR markers in respect to baseline prognostic factors alone. Reference staging system: BCLC

	Clinical			Clinical + continuous biomarker			Clinical + categorical biomarkers		
	HR	95% CI	*P* value	HR	95% CI	*p* value	HR	95% CI	*P* value
Age	1.01	(1.004–1.02)	0.005	1.01	(1.00–1.02)	0.007	1.01	(1.00–1.02)	0.004
AFP > 400 ng/ml	1.38	(1.10–1.73)	0.006	1.41	(1.12–1.77)	0.003	1.40	(1.11–1.76)	0.004
CHILD score									
A (Ref.)	1	–	–	1	–	–	1	–	–
B	1.50	(1.22–1.85)	< 0.001	1.47	(1.18–1.81)	< 0.001	1.48	(1.20–1.83)	< 0.001
C	0.97	(0.46–2.01)	> 0.9	0.93	(0.45 – 1,95)	0.9	0.91	(0.44–1.89)	0.8
BCLC stage									
Very Early (ref.)	1	–	–	1	–	–	1	–	–
Early	1.66	(1.12–2.47)	0.012	1.62	(1.09–2.41)	0.017	1.62	(1.09–2.40)	0.018
Intermediate	2.77	(1.79–4.28)	< 0.001	2.60	(1.68–4.03)	< 0.001	2.62	(1.69–4.06)	< 0.001
Advanced	4.36	(2.90–6.54)	< 0.001	4.04	(2.68–6.08)	< 0.001	4.01	(2.66–6.03)	< 0.001
Terminal	11	(5.51 – 21.8)	< 0.001	10.6	(5.32 −21)	< 0.001	10.3	(5.2 – 20.6)	< 0.001
Log (NLR)				1.33	(1.09–1.62)	0.004			
PLR				1.00	(0.99–1.00)	0.3			
CNP									
0							1	–	–
1							1.60	(1.20–2.13)	0.001
2							2.69	(1.79–4.06)	< 0.001
C-index (95% CI)	0.71 (0.68–0.73)			0.71 (0.68–0.74)			0.72 (0.68–0.74)		
IDI	Ref.			0.8% (−0.1%−2.2%, *p* = 0.07)			1.3% (0.1%, −2.7%, *p* = 0.04)		

*AFP* alpha fetoprotein, *BCLC* Barcelona clinic liver cancer, *NLR* neutrophil-to-lymphocyte ratio, *PLR* platelet-to-lymphocyte ratio, *CNP* combined NLR-PLR, *C-Index* concordance index, *IDI* integrated discrimination index, *HR* hazard ratio, *CI* confidence interval

Adding Log-NLR to the basic model did not improve the reclassification index (IDI 0.8 [−0.1–2.2%], *p* = 0.07). On the other hand, compared to the basic model, the addition of CNP significantly improves the model’s reclassification index (IDI 1.3% [0.1–2.7%], *p* = 0.04). When we included the ITA.LI.CA staging system among baseline prognostic factors, the addition of both continuous biomarkers or CNP did not improve the reclassification index (IDI 0.8 [−0.1–1.7%], *p* = 0.11 and IDI 0.8 [−0.1–2.3%], *p* = 0.06, respectively) (Supplementary Table 2). The prognostic performance of SIR markers was confirmed in the validation cohort (Supplementary Table 3 and Supplementary Table 4).

### Recurrence analysis

A subsample of 571 patients who underwent curative treatments (LT, LR, or ABL) was considered for the recurrence analysis. Two-hundred and twenty-four recurrences were recorded during the follow-up period (39%), with a median RFS of 13.9 months (95% CI 3.9–36). The 5 years recurrence rates were 3% for LT, 38% for LR, and 43% for ABL. Early recurrence rates (with a 24 month cut-off) were 41% for LR and 46.5% for ABL, whereas none of the patients who underwent LT experienced early recurrence. The median RFS for each treatment modality was as follows: 17 months [8–29] for LR, and 13 months [6–24] for ABL.

There was no significant difference in median RFS time between patients with NLR (*p* = 0.69) or PLR (*p* = 0.43) above the cut-offs and those below them. (Supplementary Fig. 3, panels *A* and *B*). Similarly, CNP was not significantly associated with RFS (*p* = 0.67) (Supplementary Fig. 3, panel *C*).

## Discussion

SIR markers are progressively gaining consensus as predictors of cancer survival, although the mechanisms by which they impact tumor biology remain unclear. Inflammatory cells, such as platelets and neutrophils, can contribute to tumor cell invasion into the peripheral blood [23]. Platelets could protect circulating tumor cells from shear stresses during circulation, induce epithelial-mesenchymal transition, and promote tumor cell extravasation to metastatic sites [24–26]. Neutrophils can enhance the adhesion and seeding of tumor cells in distant organs through the secretion of circulating growth factors [27–29]. Conversely, lymphocytes are crucial in defence against tumors, dictating the host immune response to malignancy by inducing cytotoxic cell death and inhibiting tumor cell proliferation and migration [30].

The relationship between NLR and HCC prognosis was first described by Halazun et al*.*, who demonstrated that NLR > 5 predicted poor OS and a high recurrence rate in patients undergoing LT for HCC [27]. The prognostic performance of NLR was then confirmed in HCC patients, mainly of Asian ethnicity, who received curative or palliative therapy [11, 12]. Likewise, other reports demonstrated that elevated pre-treatment PLR values predicted an unfavourable OS (HR = 1.73; 95% CI 1.46–2.04; *p* < 0.00001) and RFS (HR = 1.30; 95% CI 1.06–1.60; *p* = 0.01) irrespective of therapy [31]. Nevertheless, these biomarkers are far from routinely used in clinical practice due to the heterogeneity in study design, sample size and lack of standardized cut-off values, usually set up by the receiver operating characteristic (ROC) method. Indeed, this conventional statistical approach, widely used and easy to apply for determining an "optimal" cut-off in binary outcomes, can lead to a loss of information, reduced statistical power, and an increased risk of false positives [32].

Our study used the multivariable fractional polynomial and minimum p-value methods that considered the effect of each possible functional form of NLR and PLR, or cut-off points, for survival analysis. These approaches enabled us to identify the cut-off most strongly correlated with the outcomes (OS and RFS). By applying these cut-offs (1.45 for NLR and 188 for PLR), we could confirm that NLR and PLR effectively stratify HCC patients in terms of prognosis. Indeed, patients with biomarker values above their respective cut-off had a median OS time significantly lower than their counterparts (NLR *p* < 0.0001, and PLR *p* = 0.00015). Furthermore, at univariable and multivariable analysis adjusted for clinical covariates associated with HCC prognosis, both NLR and PLR remained independent prognostic factors for OS.

Since our cut-offs remain highly data-dependent, carrying with a serious risk of the type I error and an overestimation of the effect of the prognostic value in absolute terms, we also applied a bootstrap resampling approach that, together with a shrunk estimate, allowed us to obtain  CIs with the desired coverage. Notably, even after applying the shrinkage procedure, both NLR and PLR remained significant predictors of survival. Interestingly, when we tested the prognostic performance of CNP in our population, it was able to stratify patients into three groups with different median OS, confirming that patients with both NLR and PLR above the cut-off had a worse prognosis.

Generally, even when a biomarker has been proven to predict a disease outcome, it still needs to be demonstrated whether it can improve the survival prediction of commonly used prognostic models (such as cancer stage). Subsequently, it must be determined whether it warrants routine measurement.

The discrimination of a risk prediction model, i.e. the ability to differentiate between individuals who will experience the event of interest and those who will not, is typically assessed using the area under the ROC curve (AUC). However, the AUC has been criticized for its limited sensitivity when comparing models, especially if the baseline model already performs well [33]. In contrast, IDI is not dependent on risk categorization but considers changes in predicted risk, overcoming some of the limitations of AUC. Notably, adding CNP to the survival prediction model, including age, AFP levels, Child–Pugh score and BCLC staging, improved the IDI value (0.013, *p* = 0.04), suggesting that CNP significantly enhanced the model’s ability to predict OS.

Regarding HCC recurrence, literature data on the prognostic significance of NLR and PLR are somewhat controversial. Heterogeneity in study design, variability in sample size and characteristics of enrolled populations, and lack of standardised cut-off values of SIR biomarkers can account for the discrepancy in the results [34]. In our study, neither NLR nor PLR significantly predicted HCC recurrence. There are several potential reasons for the lack of association between SIR markers and RFS. The retrospective design of the study introduces the risk of information bias, despite efforts to minimise this. Additionally, our cohort had more favorable tumor characteristics, with a higher number of patients having Child–Pugh class A or B, a single nodule, and a median tumor diameter of less than 50 mm. Furthermore, SIR markers may reflect other aspects of disease that are not directly related to cancer, and therefore may not accurately predict HCC recurrence.

In recent years, the approval of antiprogrammed cell death-1 (PD-1) antibodies, which act as immune checkpoint inhibitors (ICIs), has revolutionized the treatment landscape for HCC. We have not had long-term observations of patients undergoing ICI therapy. However, a recent pooled meta-analysis of 44 studies involving 5,322 patients confirmed the predictive value of baseline SIR biomarkers, such as NLR, for OS also in HCC patients receiving ICI treatment (HR1.95, *p* < 0.001) [35].

We are aware that some shortcomings may have influenced our study. First, this is a retrospective study, so a potential selection bias and some unintended biases are predictable. Second, we lack external validation although the bootstrap resampling method used in the training set and the internal validation, at least in part, overcome this limitation, allowing us to establish a stable prognostic "multiparametric" model taking tumor burden, liver function, and inflammatory status into account. The CRP is not routinely taken for examination in the treatment of HCC patients. Lastly, since neutrophil, platelet, and lymphocyte levels are influenced by infections, inflammation in other tissues, and medications taken before HCC treatment, these factors should be considered when interpreting NLR and PLR measurements.

On the other hand, we believe that including a large cohort of HCC patients managed with different strategies, extended follow-up, and a rigorous statistical approach strengthens our results. Indeed, to the best of our knowledge, this is the largest Western series of HCC patients in whom the reliability of pre-treatment SIR markers in predicting OS and RFS has been rigorously tested and validated.

In conclusion, our study showed that NLR, PLR, and their combination, CNP, are reliable predictors of prognosis in patients with HCC, enhancing the accuracy of traditional factors like cancer stage and liver function. Thus, due to their non-invasiveness, ease of determination, repeatability, and low cost, these biomarkers are strong candidates for improving prognosis prediction in HCC patients. External validation by prospective and well-powered studies would be needed before their routine adoption in clinical practice.

## Supplementary Information

Below is the link to the electronic supplementary material.

Supplementary file1 (DOCX 529 KB)

Supplementary file2 (DOCX 33 KB)

## Data Availability

The data that support the findings of this study are available from the corresponding author upon reasonable request.

## References

[1] Sung H, Ferlay J, Siegel RL, Laversanne M, Soerjomataram I, Jemal A, et al. Global cancer statistics 2020: GLOBOCAN estimates of incidence and mortality worldwide for 36 cancers in 185 countries. CA Cancer J Clin. 2021;71:209–249 33538338 10.3322/caac.21660

[2] Galle PR, Forner A, Llovet JM, Mazzaferro V, Piscaglia F, Raoul JL, et al. EASL clinical practice guidelines: management of hepatocellular carcinoma. J Hepatol. 2018;69:182–236 29628281 10.1016/j.jhep.2018.03.019

[3] Vitale A, Farinati F, Finotti M, Di Renzo C, Brancaccio G, Piscaglia F, et al. (2021) Overview of Prognostic Systems for Hepatocellular Carcinoma and ITA.LI.CA External Validation of MESH and CNLC Classifications. Cancers (Basel). 13:1673. Available from: https://www.mdpi.com/2072-6694/13/7/1673 10.3390/cancers13071673 PMC8037197 33918125

[4] Galle PR, Foerster F, Kudo M, Chan SL, Llovet JM, Qin S, et al. (2019) Biology and significance of alpha‐fetoprotein in hepatocellular carcinoma. Liver Int. 39:2214–29. Available from: https://onlinelibrary.wiley.com/doi/10.1111/liv.14223 10.1111/liv.14223 31436873

[5] Hanahan D, Weinberg RA (2011) Hallmarks of cancer: the next generation. Cell. 144:646–74. Available from: http://www.ncbi.nlm.nih.gov/pubmed/21376230 10.1016/j.cell.2011.02.013 21376230

[6] Ferrucci PF, Gandini S, Battaglia A, Alfieri S, Di Giacomo AM, Giannarelli D, et al. Baseline neutrophil-to-lymphocyte ratio is associated with outcome of ipilimumab-treated metastatic melanoma patients. Br J Cancer. 2015;112:1904–1910 26010413 10.1038/bjc.2015.180 PMC4580390

[7] Grivennikov SI, Greten FR, Karin M. Immunity, inflammation, and cancer. Cell. 2010;140:883–899 20303878 10.1016/j.cell.2010.01.025 PMC2866629

[8] Koh C-H, Bhoo-Pathy N, Ng K-L, Jabir RS, Tan G-H, See M-H, et al. Utility of pre-treatment neutrophil–lymphocyte ratio and platelet–lymphocyte ratio as prognostic factors in breast cancer. Br J Cancer. 2015;113:150–158 26022929 10.1038/bjc.2015.183 PMC4647546

[9] Halazun KJ, Hardy MA, Rana AA, Woodland DC, Luyten EJ, Mahadev S, et al. (2009) Negative Impact of Neutrophil-Lymphocyte Ratio on Outcome After Liver Transplantation for Hepatocellular Carcinoma. Ann Surg. 250:141–51. Available from: https://journals.lww.com/00000658-200907000-00022 10.1097/SLA.0b013e3181a77e59 19561458

[10] Gomez D, Farid S, Malik HZ, Young AL, Toogood GJ, Lodge JPA, et al. Preoperative neutrophil-to-lymphocyte ratio as a prognostic predictor after curative resection for hepatocellular carcinoma. World J Surg. 2008;32:1757–1762 18340479 10.1007/s00268-008-9552-6

[11] Ji F, Liang Y, Fu S-J, Guo Z-Y, Shu M, Shen S-L, et al. (2016) A novel and accurate predictor of survival for patients with hepatocellular carcinoma after surgical resection: the neutrophil to lymphocyte ratio (NLR) combined with the aspartate aminotransferase/platelet count ratio index (APRI). BMC Cancer. 16:137. Available from: http://www.ncbi.nlm.nih.gov/pubmed/26907597 10.1186/s12885-016-2189-1 PMC4763424 26907597

[12] Huang ZL, Luo J, Chen MS, Li JQ, Shi M. Blood neutrophil-to-lymphocyte ratio predicts survival in patients with unresectable hepatocellular carcinoma undergoing transarterial chemoembolization. J Vasc Interv Radiol. 2011;22:702–709 21514523 10.1016/j.jvir.2010.12.041

[13] Altman DG, Lausen B, Sauerbrei W, Schumacher M. Dangers of using “optimal” cutpoints in the evaluation of prognostic factors. JNCI J Natl Cancer Inst. 1994;86:829–835. 10.1177/1010428317707375 8182763 10.1093/jnci/86.11.829

[14] Xue T-C, Jia Q-A, Ge N-L, Zhang B-H, Wang Y-H, Ren Z-G, et al. The platelet-to-lymphocyte ratio predicts poor survival in patients with huge hepatocellular carcinoma that received transarterial chemoembolization. Tumor Biol. 2015;36:6045–6051 10.1007/s13277-015-3281-x 25731733

[15] EASL Clinical Practice Guidelines. Management of hepatitis C virus infection. J Hepatol. 2011;55:245–264 21371579 10.1016/j.jhep.2011.02.023

[16] Mutimer D, Aghemo A, Diepolder H, Negro F, Robaeys G, Ryder S, et al. EASL clinical practice guidelines: management of hepatitis C virus infection. J Hepatol. 2014;60:392–420 24331294 10.1016/j.jhep.2013.11.003

[17] Practice Guidelines Panel C, Pawlotsky J-M, Board G, representative E, Negro F, members P, Aghemo A, et al. EASL recommendations on treatment of hepatitis C: final update of the series q European Association for the Study of the Liver*. J Hepatol. 2020;73:1170–1218 32956768 10.1016/j.jhep.2020.08.018

[18] Lampertico P, Agarwal K, Berg T, Buti M, Janssen HLA, Papatheodoridis G, et al. EASL 2017 clinical practice guidelines on the management of hepatitis B virus infection. J Hepatol. 2017;67:370–398 28427875 10.1016/j.jhep.2017.03.021

[19] Papatheodoridis G, Buti M, Cornberg M, Janssen H, Mutimer D, Pol S, et al. EASL clinical practice guidelines: Management of chronic hepatitis B virus infection. J Hepatol. 2012;57:167–185 22436845 10.1016/j.jhep.2012.02.010

[20] Reig M, Forner A, Rimola J, Ferrer-Fàbrega J, Burrel M, Garcia-Criado Á, et al. BCLC strategy for prognosis prediction and treatment recommendation. J Hepatol. 2022. 10.1016/j.jhep.2021.11.018 34801630 10.1016/j.jhep.2021.11.018 PMC8866082

[21] Farinati F, Vitale A, Spolverato G, Pawlik TM, Huo T, Lee Y-H, et al. Development and validation of a new prognostic system for patients with hepatocellular carcinoma. PLOS Med. 2016;13:e1002006. 10.1371/journal.pmed.1002006 27116206 10.1371/journal.pmed.1002006 PMC4846017

[22] Holländer N, Sauerbrei W, Schumacher M. Confidence intervals for the effect of a prognostic factor after selection of an “optimal” cutpoint. Stat Med. 2004;23:1701–1713 15160403 10.1002/sim.1611

[23] Hanahan D, Weinberg RA. Hallmarks of cancer: the next generation. Cell. 2011;144:646–674 21376230 10.1016/j.cell.2011.02.013

[24] Hanahan D, Weinberg RA (2011) Hallmarks of Cancer: The Next Generation. Cell. 144:646–74. Available from: https://linkinghub.elsevier.com/retrieve/pii/S0092867411001279 10.1016/j.cell.2011.02.013 21376230

[25] Labelle M, Begum S, Hynes RO. Direct signaling between platelets and cancer cells induces an epithelial-mesenchymal-like transition and promotes metastasis. Cancer Cell. 2011;20:576–590 22094253 10.1016/j.ccr.2011.09.009 PMC3487108

[26] Holländer N, Sauerbrei W, Schumacher M. Confidence intervals for the effect of a prognostic factor after selection of an “optimal” cutpoint. Stat Med. 2004;23:1701–13. Available from: http://www.ncbi.nlm.nih.gov/pubmed/15160403 10.1002/sim.1611 15160403

[27] Halazun KJ, Hardy MA, Rana AA, Woodland DC IV, Luyten EJ, Mahadev S, et al. Negative impact of neutrophil-lymphocyte ratio on outcome after liver transplantation for hepatocellular carcinoma. Ann Surg. 2009;250:141–151 19561458 10.1097/SLA.0b013e3181a77e59

[28] Dan J, Zhang Y, Peng Z, Huang J, Gao H, Xu L, et al. Postoperative neutrophil-to-lymphocyte ratio change predicts survival of patients with small hepatocellular carcinoma undergoing radiofrequency ablation. PLoS ONE. 2013. 10.1371/journal.pone.0058184 23516447 10.1371/journal.pone.0058184 PMC3597630

[29] Cools-Lartigue J, Spicer J, McDonald B, Gowing S, Chow S, Giannias B, et al. (2013) Neutrophil extracellular traps sequester circulating tumor cells and promote metastasis. J Clin Invest. 123:3446–58. Available from: http://www.jci.org/articles/view/67484 10.1172/JCI67484 PMC3726160 23863628

[30] Placke T, Salih HR, Kopp H-G (2012) GITR ligand provided by thrombopoietic cells inhibits NK cell antitumor activity. J Immunol. 189:154–60. Available from: https://www.nature.com/articles/nature07205 10.4049/jimmunol.1103194 22649191

[31] Lin W, Zhong M, Zhang Y, Wang H, Zhao H, Cheng B, et al. Prognostic role of platelet-to-lymphocyte ratio in hepatocellular carcinoma with different BCLC stages: a systematic review and meta-analysis. Gastroenterol Res Pract. 2018;2018:1–10 10.1155/2018/5670949 PMC6109515 30158964

[32] Altman DG, Lausen B, Sauerbrei W, Schumacher M. Dangers of using “optimal” cutpoints in the evaluation of prognostic factors. JNCI J Natl Cancer Inst. 1994;86:829–835 8182763 10.1093/jnci/86.11.829

[33] Liu Y, Wang Z-X, Cao Y, Zhang G, Chen W-B, Jiang C-P (2016) Preoperative inflammation-based markers predict early and late recurrence of hepatocellular carcinoma after curative hepatectomy. Hepatobiliary Pancreat Dis Int. 15:266–74. Available from: http://www.ncbi.nlm.nih.gov/pubmed/27298102 10.1016/s1499-3872(16)60094-2 27298102

[34] Mouchli M, Reddy S, Gerrard M, Boardman L, Rubio M (2021) Usefulness of neutrophil-to-lymphocyte ratio (NLR) as a prognostic predictor after treatment of hepatocellular carcinoma." Review article. Ann Hepatol. 22:100249. Available from: http://www.ncbi.nlm.nih.gov/pubmed/32896610 10.1016/j.aohep.2020.08.067 32896610

[35] Zhang L, Feng J, Kuang T, Chai D, Qiu Z, Deng W, et al. Blood biomarkers predict outcomes in patients with hepatocellular carcinoma treated with immune checkpoint inhibitors: a pooled analysis of 44 retrospective sudies. Int Immunopharmacol. 2023;118:110019 36933492 10.1016/j.intimp.2023.110019

