# Supplementary material for: Systemic inflammatory response markers improve the discrimination for prognostic model in hepatocellular carcinoma
Source: Hepatol Int. 2025 Mar 25;19(4):915–28. doi: 10.1007/s12072-025-10806-6 (PMC12287231; doi:10.1007/s12072-025-10806-6)
Supplement: Supplementary file 1 — Supplementary file1 (DOCX 529 KB) [file 12072_2025_10806_MOESM1_ESM.docx]

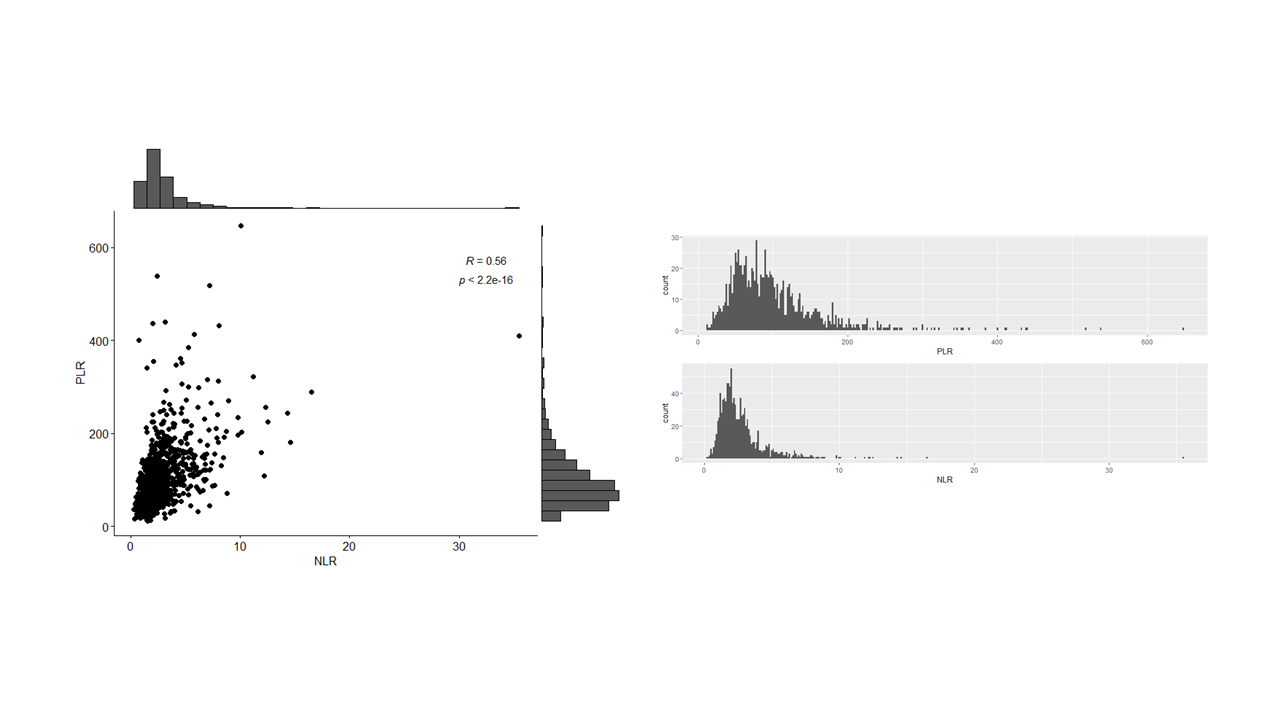


**Supplementary Figure 1.** Histograms showing the distribution of NLR and PLR in the whole cohort of HCC patients (n = 1,043) (right side) and scatter diagram showing the positive correlation of NLR with PLR (r = 0.55, p < 0.001) (left side).

**
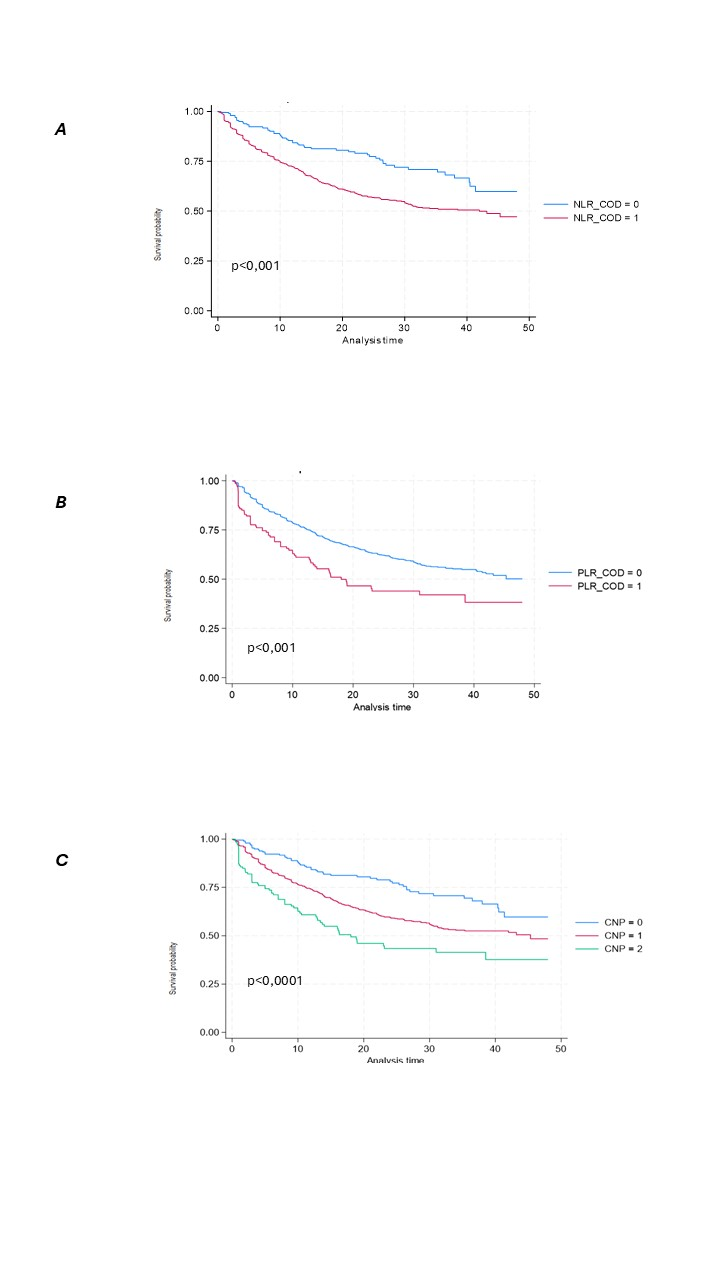
**

**Supplementary Figure 2**. Kaplan-Meier curves of survival data of HCC patients (n = 1,243) according to NLR (a) and PLR (b) best cut-offs and CNP (c). Time on the x-axis represents months of observation (follow-up extended up to 206 months). P value is a log-rank test.

**
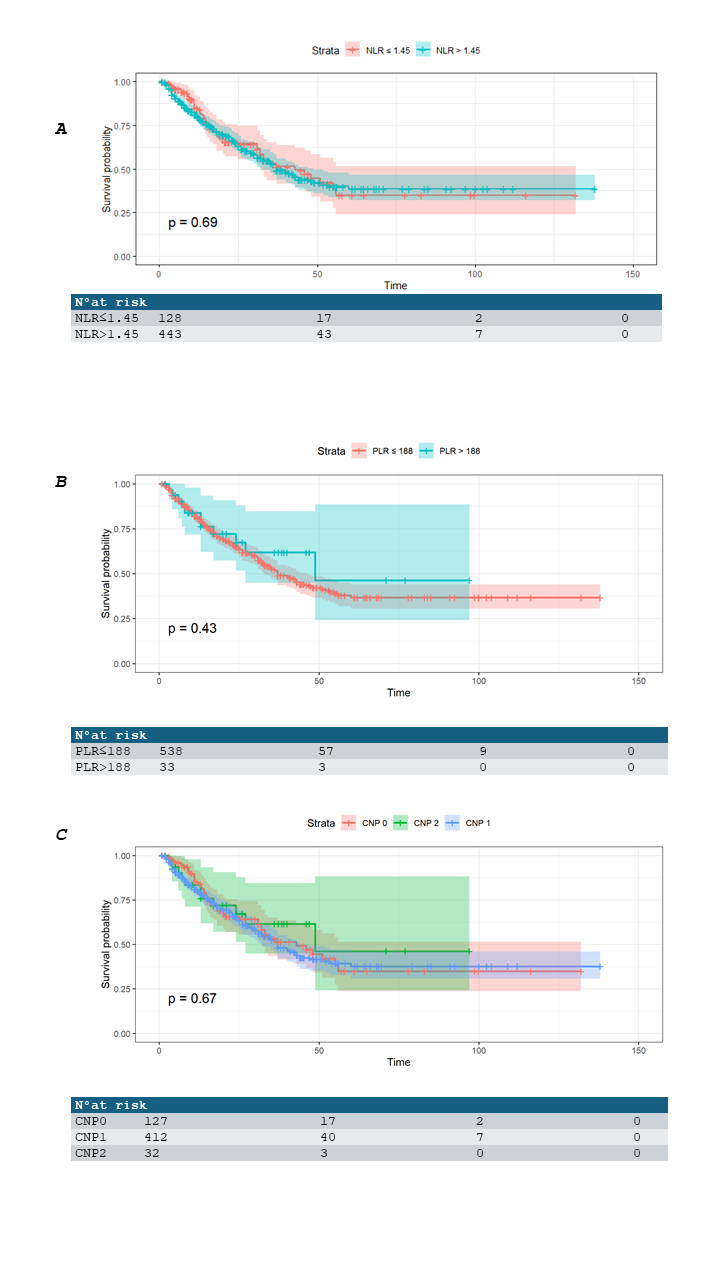
**

**Supplementary Figure 3.** Kaplan-Meier curves of recurrence data in HCC patients (n = 571) according to NLR (a) and PLR (b) best cut-off and CNP (c). Time on the x-axis represents months of observation (follow-up extended up to 137 months). The table with subjects at risk is reported for each biomarker at each specific time point. P value is a log-rank test.
