# Supplementary material for: Systemic inflammatory response markers improve the discrimination for prognostic model in hepatocellular carcinoma
Source: Hepatol Int. 2025 Mar 25;19(4):915–28. doi: 10.1007/s12072-025-10806-6 (PMC12287231; doi:10.1007/s12072-025-10806-6)
Supplement: Supplementary file 2 — Supplementary file2 (DOCX 33 KB) [file 12072_2025_10806_MOESM2_ESM.docx]

**Supplementary Table 1.** Association between NLR and PLR and clinical prognostic factors

|  | **NLR**  **(median [IQR])** | **p** | **PLR**  **(median [IQR])** | **p** |
| --- | --- | --- | --- | --- |
| **Age** | 0.015(-0.04-0.07) | 0.6173^1^ | 0.15(0.09-0.20) | <0.001^1^ |
|  |  |  |  |  |
| **AFP (ng/mL)** |  |  |  |  |
| **>400** | 2.17 [1.60-3.20] | 0.400 | 84 [56-119] | 0.002 |
| **<400** | 2.34 [1.72-3.11] |  | 96 [65-133] |  |
| **Child-Pugh score** |  |  |  |  |
| **A** | 2.12 [1.50-3.00] | <0.001^2^ | 89 [61-124] | 0.300^2^ |
| **B** | 2.53 [1.75-3.68] |  | 86 [54-123] |  |
| **C** | 2.66 [1.85-4.57] |  | 67 [49-113] |  |
|  |  |  |  |  |
| **BCLC stage** |  |  |  |  |
| **0** | 1,99 [1.37-2.75] | <0.001^2^ | 78 [59-112] | <0.001^2^ |
| **A** | 2.08 [1.52-2.98] |  | 79 [55-113] |  |
| **B** | 2.19 [1.68-3.15] |  | 92 [63-135] |  |
| **C** | 2.65 [1.81-3.83] |  | 94 [66-137] |  |
| **D** | 2.59 [1.84-3.80] |  | 81 [56-120] |  |

*^1^ Pearson correlation; ^2^ Kruskal-Wallis rank sum test.*

*NLR: neutrophil-to-lymphocyte ratio; PLR: platelet-to-lymphocyte ratio; AFP: alpha-fetoprotein; BCLC: Barcelona Clinic Liver Cancer*

**Supplementary Table 2:**  Comparison of discrimination for a new model including baseline prognostic factors and SIR markers in respect to baseline prognostic factors alone. Reference staging system: ITA.Li.CA

|  | **Clinical** | | | **Clinical + continuous biomarker** | | | **Clinical + Categorical Biomarkers** | | |
| --- | --- | --- | --- | --- | --- | --- | --- | --- | --- |
|  | HR | 95% CI | *P* value | HR | 95% CI | *P* value | HR | 95% CI | *P* value |
|  |  |  |  |  |  |  |  |  |  |
| **Age** | 1.02 | 1.01, 1.03 | 0.002 | 1.02 | 1.00, 1.03 | 0.005 | 1.02 | 1.01, 1.03 | 0.004 |
|  |  |  |  |  |  |  |  |  |  |
| **AFP >400 ng/ml** | 1.34 | 1.05, 1.73 | 0.020 | 1.37 | 1.06, 1.76 | 0.014 | 1.38 | 1.07, 1.77 | 0.012 |
|  |  |  |  |  |  |  |  |  |  |
| **CHILD score** |  |  |  |  |  |  |  |  |  |
| **A** | — | — |  | — | — |  | — | — |  |
| **B** | 1.82 | 1.46, 2.28 | <0.001 | 1.85 | 1.48, 2.32 | <0.001 | 1.84 | 1.47, 2.30 | <0.001 |
| **C** | 3.70 | 2.13, 6.42 | <0.001 | 3.76 | 2.16, 6.55 | <0.001 | 3.34 | 1.91, 5.82 | <0.001 |
|  |  |  |  |  |  |  |  |  |  |
| **ITA.LI.CA stage** |  |  |  |  |  |  |  |  |  |
| **0** | — | — |  | — | — |  | — | — |  |
| **A** | 1.40 | 1.01, 1.92 | 0.041 | 1.36 | 0.99, 1.88 | 0.056 | 1.36 | 0.99, 1.88 | 0.058 |
| **B** | 2.25 | 1.60, 3.16 | <0.001 | 2.12 | 1.50, 2.99 | <0.001 | 2.15 | 1.53, 3.04 | <0.001 |
| **C** | 8.62 | 6.03, 12.3 | <0.001 | 8.03 | 5.60, 11.5 | <0.001 | 8.01 | 5.58, 11.5 | <0.001 |
|  |  |  |  |  |  |  |  |  |  |
| **Log(NLR)** |  |  |  | 1.00 | 1.00, 1.00 | 0.2 |  |  |  |
| **PLR** |  |  |  | 1.19 | 0.95, 1.50 | 0.13 |  |  |  |
| **CNP** |  |  |  |  |  |  |  |  |  |
| **0** |  |  |  |  |  |  | 1 | - | - |
| **1** |  |  |  |  |  |  | 1.30 | 0.97, 1.75 | 0.080 |
| **2** |  |  |  |  |  |  | 2.24 | 1.45, 3.46 | <0.001 |
| **C-Index (95% CI)** | 0.73 (0.69-0.75) | | | 0.73 (0.69-0.76) | | | 0.734 (0.70-0.76) | | |
| **IDI** | ref. | | | 0.8% (-0.1%-1.7%, p=0.11) | | | 0.8% (-0.1%, -2.3%, p=0.06) | | |

*AFP: alpha-fetoprotein; ITA.Li.Ca: Italian Liver Cancer; NLR: neutrophil-to-lymphocyte ratio; PLR: platelet-to lymphocyte ratio; CNP: combined NLR-PLR; C-Index: concordance index; IDI: integrated discrimination index; HR = Hazard Ratio; CI = Confidence Interval.*

**Supplementary Table 3.** Comparison of discrimination for a new model including baseline prognostic factors and SIR markers in respect to baseline prognostic factors alone in the validation cohort. Reference staging system: BCLC.

|  | **Clinical** | | | **Clinical + Continuous biomarker** | | | **Clinical + Categorical Biomarkers** | | |
| --- | --- | --- | --- | --- | --- | --- | --- | --- | --- |
|  | HR | 95% CI | *P* value | HR | 95% CI | *P* value | HR | 95% CI | *P* value |
| **Age** | 1.00 | 0.99,1.01 | 0.4 | 1.00 | 0.99,1.01 | 0.292 | 1.00 | 0.99,1.01 | 0.432 |
| **AFP >400 ng/ml** | 2.68 | 2.13,3.38 | <0.001 | 2.59 | 2.05,3.26 | <0.001 | 2.55 | 2.02,3.21 | <0.001 |
|  |  |  |  |  |  |  |  |  |  |
| **CHILD score** |  |  |  |  |  |  |  |  |  |
| **A (ref.)** | — | — |  | — |  |  |  |  |  |
| **B** | 10.3 | 0.81,1.32 | 0.751 | 1.02 | 0.80,1.30 | 0.857 | 1.03 | 0.81,1.32 | 0.773 |
| **C** | 1.36 | 0.79,1.40 | 0.253 | 1.28 | 0.75,2.20 | 0.363 | 1.30 | 0.77,2.21 | 0.314 |
|  |  |  |  |  |  |  |  |  |  |
| **BCLC stage** |  |  |  |  |  |  |  |  |  |
| **Very Early (ref.)** | 1 | — | — |  |  |  | 1 | - | - |
| **Early** | 0.88 | 0.55,1.4 | 0.601 | 0.86 | 0.54,1.37 | 0.533 | 0.87 | 0.54,1.38 | 0.563 |
| **Intermediate** | 0.93 | 0.54,1.60 | 0.802 | 0.92 | 0.53,1.59 | 0.766 | 0.93 | 0.54,1.60 | 0.801 |
| **Advanced** | 1.21 | 0.75,1.95 | 0.438 | 1.19 | 0.74,1.93 | 0.473 | 1.17 | 0.72,1.90 | 0.508 |
| **Terminal** | 1.12 | 0.57,2.16 | 0.741 | 1.12 | 0.57,2.18 | 0.728 | 1.12 | 0.58,2.16 | 0.726 |
| **Log (NLR)** |  |  |  | 1.76 | 1.04,1.10 | <0.001 |  |  |  |
| **PLR** |  |  |  | 0.99 | 0.70,1.05 | 0.447 |  |  |  |
| **CNP** |  |  |  |  |  |  |  |  |  |
| **0** |  |  |  |  |  |  | 1 | - | - |
| **1** |  |  |  |  |  |  | 1.66 | 1.20,2.30 | 0.002 |
| **2** |  |  |  |  |  |  | 2.21 | 1.46,3.34 | <0.001 |
| **C-Index (95% CI)** | 0.63 (0.60 – 0.66) | | | 0.65 (0.62 – 0.68) | | | 0.65 (0.62-0.68) | | |

*AFP: alpha fetoprotein; BCLC Barcelona Clinic Liver Cancer; NLR: neutrophil-to-lymphocyte ratio; PLR: platelet-to-lymphocyte ratio; CNP: combined NLR-PLR; HR = Hazard Ratio, CI = Confidence Interval*

**Supplementary Table 4:**  Comparison of discrimination for a new model including baseline prognostic factors and SIR markers in respect to baseline prognostic factors alone in the validation cohort. Reference staging system: ITA.Li.CA

|  | **Clinical** | | | **Clinical + continuous biomarker** | | | **Clinical + Categorical Biomarkers** | | |
| --- | --- | --- | --- | --- | --- | --- | --- | --- | --- |
|  | HR | 95% CI | *P* value | HR | 95% CI | *P* value | HR | 95% CI | *P* value |
| **Age** | 1.00 | 0.99,1.01 | 0.504 | 1.00 | 0.99,1.01 | 0.339 | 1.00 | 0.99,1.01 | 0.543 |
| **AFP >400 ng/ml** | 1.99 | 1.57,2.53 | <0.001 | 1.96 | 1.66,2.68 | <0.001 | 1.94 | 1.52,2.46 | <0.001 |
| **CHILD score** |  |  |  |  |  |  |  |  |  |
| **A** | — | — |  | — | — |  | — | — |  |
| **B** | 1.11 | 0.88,1.40 | 0.343 | 1.09 | 0.89,1.42 | 0.426 | 1.11 | 0.88,1.40 | 0.371 |
| **C** | 1.43 | 0.94,2.16 | 0.088 | 1.45 | 0.94,2.15 | 0.077 | 1.41 | 0.93,2.13 | 0.098 |
|  |  |  |  |  |  |  |  |  |  |
| **ITA.LI.CA stage** |  |  |  |  |  |  |  |  |  |
| **0** | — | — |  | — | — |  | — | — |  |
| **A** | 1.40 | 0.95,2.08 | 0.087 | 1.37 | 1.00,2.22 | 0.114 | 1.39 | 0.94,2.06 | 0.094 |
| **B** | 3.34 | 2.32,4.79 | <0.001 | 3.13 | 2.51,5.24 | <0.001 | 3.16 | 2.19,4.55 | <0.001 |
| **C** | 3.16 | 2.10,4.74 | <0.001 | 2.99 | 2.26,5.13 | <0.001 | 3.05 | 2.03,4.59 | <0.001 |
|  |  |  |  |  |  |  |  |  |  |
| **Log(NLR)** |  |  |  | 1.68 | 0.60,0.90 | <0.001 |  |  |  |
| **PLR** |  |  |  | 0.99 | 1.06,1.12 | 0.218 |  |  |  |
| **CNP** |  |  |  |  |  |  |  |  |  |
| **0** |  |  |  |  |  |  | 1 | - |  |
| **1** |  |  |  |  |  |  | 1.45 | 1.04,2.01 | 0.025 |
| **2** |  |  |  |  |  |  | 1.76 | 1.16,2.67 | 0.007 |
| **C-Index (95% CI)** | 0.68 (0.66-0.72) | | | 0.69 (0.67-0.72) | | | 0.69 (0.67-0.72) | | |

*AFP: alpha-fetoprotein; ITA.Li.Ca: Italian Liver Cancer; NLR: neutrophil-to-lymphocyte ratio; PLR: platelet-to lymphocyte ratio; CNP: combined NLR-PLR; HR = Hazard Ratio; CI = Confidence Interval.*
